# Empirical Research into the Development Mechanism of Industry Innovation of Health and Wellness Tourism in the Context of the Sharing Economy

**DOI:** 10.3390/ijerph191912479

**Published:** 2022-09-30

**Authors:** Li Li, Xuejun Chen

**Affiliations:** 1 School of Tourism and Service Management, Chongqing University of Education, Chongqing 400065, China; 2 School of Literature, Chongqing Jiaotong University, Chongqing 400074, China

**Keywords:** health and wellness tourism, industry sharing, sharing economy, industry innovation

## Abstract

Health and wellness tourism has become an emerging form of tourism economy. In the era of the sharing economy, it is of theoretical and practical significance to study the development mechanism of the industry innovation (II) of health and wellness tourism. Based on theoretical analysis, hypotheses were proposed for the relationships of industry sharing (IS) as well as its constitutional dimensions with the resource acquisition capability (RAC), policy support (PS), and II of health and wellness tourism. The constitutional dimensions of IS include public operational resources (PORs), infrastructures, and industry cooperation networks (ICNs). In addition, a model for the influencing mechanism of IS on the II of health and wellness tourism was established. Questionnaire surveys were conducted with 542 managers of health and wellness tourism enterprises in 21 provinces (or municipalities) in China, using the empirical research method, and the survey data are subjected to mathematical statistical analysis. Results show that the PORs, infrastructures, and ICNs of the IS of health and wellness tourism have significant positive influences on the II. RAC has a partial mediating effect between the IS and the II of health and wellness tourism. PS exerts a moderating effect between the IS and the II of health and wellness tourism. Finally, suggestions for promoting the II development of health and wellness tourism are proposed from the perspective of optimizing IS.

## 1. Introduction

The development of the industry innovation (II) of health and wellness tourism is a complicated system, working under the comprehensive action of many influencing factors, when overviewing the development course of the industry in China and abroad. The II development of health and wellness tourism is a dynamic process, which is adjusted according to the external and internal environments. In the new era, the sharing economy has risen and exerted revolutionary influences on traditional health and wellness tourism in terms of industrial ecology, mode of development, and business logic. In addition, it has brought new challenges and a set of new requirements for the development of health and wellness tourism. The development mechanism of the II of health and wellness tourism, in the context of the sharing economy, has changed substantially. What are the key factors that influence the II development of health and wellness tourism in the new environment? How do these key factors influence the II development of health and wellness tourism under the action of the external environment? To answer these questions, this research explores the mechanism of the II development of health and wellness tourism from the perspective of the sharing economy. A theoretical model for the II development mechanism of the industry, in the context of sharing economy, is established based on a normative study. Moreover, the empirical research method is used to verify the model, which is conducive to enriching and expanding the II theory of tourism. The research provides a new theoretical tool for the II development of health and wellness tourism. In addition, it also addresses practical problems in the industry from the practical perspective, so it is of important theoretical and practical significance.

## 2. Literature Review and Hypotheses

### 2.1. Literature Review

An Austrian American economist, Joseph Alois Schumpeter, introduced the concept of “innovation” into economics in 1912 and laid a theoretical basis for modern innovation in his representative writing, The Theory of Economic Development [1]. Since then, industry innovation (II) has become one of the important aspects of research into the industrial economy. Research on the II in tourism can be dated back to the 1990s, and it remains a novel research area. Scholars across the world have conducted numerous theoretical and empirical studies on the II mechanism of tourism. Due to the comprehensive features of tourism, the II development of tourism is under the joint influences of multiple factors. Some scholars took the industry boundary as a classification standard to divide influencing factors into external and internal ones or macroscopic and microscopic ones. These factors include enterprise scale, enterprise management mode, human capital and entrepreneurship, institution and policy, enterprise cooperation and agglomeration, market demand, input in technological innovation, economic development level, innovation resources, knowledge base, and government policy [2,3,4,5].

As the research deepens, influencing factors in relevant studies in China and abroad are refined; that is, these studies explore the influences of one or more factors on the II in tourism. The dual demands of people for health and tourism have driven the new market demand for health and wellness tourism to grow rapidly. Changes in market demands of tourism and consumption upgrading can promote innovation of products, optimize operation management, develop new markets, and drive the II development of tourism [6,7]. Technical progress is the basis for, and driving force behind, changing the industrial structure of tourism. Technologies, particularly information technology (IT), have been widely used for tourism and have exerted significant influences on tourism in many aspects. These include innovating products, improving operating and management efficiency, optimizing procedures, and reforming organizations. These technologies lead and drive the II development of tourism [8]. Cooperative ability is the combination of relational capability, alliance capability, and network capability. Cooperative ability can motivate the proactive behaviors of innovation networks, realize the constant upgrading of innovation networks, and, therefore, drive the sustainable development of corresponding industries [9]. The cooperation and agglomeration of enterprises help enterprises to obtain external innovation resources, bring advantages of resource agglomeration and economies of scale, and, thus, improve innovation performance [10]. Human capital plays an important part in the innovation of tourism. Sophisticated enterprise managers and staff can absorb external knowledge, identify market opportunities, fulfill internal innovation, improve the management level and innovation ability, and facilitate innovation among tourism enterprises [11]. Knowledge is one of the most important innovation resources. Knowledge sharing is beneficial for knowledge and information transfer in enterprises; information and knowledge resources are conducive to the improvement of the learning ability and innovation ability of members in the industry, thus promoting II [12,13].

In summary, existing research results are characterized by the constant expansion of the research scope and the gradual refinement and deepening of the research content. This reflects that relevant research has a wide prospect and some limitations. The key influencing factors for the II development of health and wellness tourism remain in dispute, so a unified understanding has not been reached. In addition, there remains a lack of comprehensive, in-depth understanding of the II process and mechanism of health and wellness tourism. In addition, the influencing mechanisms of mediating and moderating variables remain unclear. Given the cross-cultural background, the introduction of new moderating variables is a new trend of research into the II in health and wellness tourism. However, moderating variables of the II in the Chinese context and the industrial situation of health and wellness tourism are rarely studied. In brief, the lag in theoretical and practical research has restricted the development of health and wellness tourism in China, making it necessary to strengthen the relevant research.

### 2.2. Hypotheses

#### 2.2.1. Influences of Industry Sharing on Resource Acquisition Capability

The strategic target of industry sharing (IS) of health and wellness tourism in the sharing economy is to construct intra-industry and inter-industry resource sharing systems. Apart from this, it also aims to improve the identification, collection, use, and allocation efficiency of resources in industrial operation, thus enhancing the resource acquisition capability (RAC) and overall resource allocation efficiency in the industry.

IS can gather all kinds of factors and conditions needed for the industrial development and innovation, and these public goods may benefit each member in the industry (Olson, 1993) [14]. IS can produce spillover effects in the generic technology and production system and attract high-quality resources and factors conducive to industrial development to agglomerate, including high-quality talent, large enterprises, and technical infrastructures. Health and wellness tourism resources include forest, water, climate, countryside, culture, and medical resources. Tourism resources, as the object of tourism activities and basic conditions for tourism development, are conducive to the agglomeration of other production factors [15]. In the context of the sharing economy, the IS of health and wellness tourism enables the flow and exchange of all types of resources including information, knowledge, materials, and capital between the inside and outside of the industry [16]. In addition, IS uses systematic and scientific management methods for allocation optimization and the reintegration of resource factors, thus influencing the RAC.

Infrastructure development increases in pace with industrial output, so constructing and improving infrastructures is the premise for the development of health and wellness tourism. Health and wellness tourism features favorable municipal infrastructures, medical facilities, cultural and sports facilities, commercial service facilities, rescue facilities, and public information facilities [17]. These facilities are not only beneficial to the development of the industry but also favorable for creating a benign investment environment and attracting more capital investment and the agglomeration of strong enterprises. The shared network platforms established using several technical means, in the context of the sharing economy, innovate the resource allocation mode and, therefore, improve the overall resource utilization efficiency of the industry. These technical means include mobile Internet technologies, big data, cloud computing, and intelligent terminals. The shared network platforms enable health and wellness tourism enterprises to share public technological platforms, public knowledge, marketing channels, labor markets and services, and public facilities [18]. This allows the enterprises to obtain external resources rapidly, economically, and conveniently and realize the sharing and integration of internal and external innovation resources, thus improving the RAC in the industry.

The industrial chain of health and wellness tourism is a chain constituted by all value formation and transfer links from the lowest input of raw materials to the formation of final products. It includes the value chains of tourism suppliers, tourism channels, and tourism consumers. Tourism enterprises in each link of the industrial chain are connected through contracts and transaction relationships, thus forming a crisscross social network. The stronger these social networks are, then more channels are available for enterprises to obtain resources, the relationships are closer between members, and the amount of resources exchanged is greater, which improves the RAC of enterprises [19]. Building a solid relationship with suppliers helps health and wellness tourism enterprises to obtain more effective market information, raw materials and services of higher quality, wider funding of turnover space, and a more preferential price. It also enables sharing of technological support and management experience [20]. If health and wellness tourism enterprises establish a good relationship with competing enterprises, it contributes to the communication between them and is of benefit to resource sharing [21]. Building an effective cooperative relationship with downstream marketing enterprises helps health and wellness tourism enterprises to master, in a timely manner, both market dynamics and customer demand. It also improves the ability of these enterprises to acquire information resources through cooperation and information sharing [22]. Based on the above analysis, the following hypotheses are proposed:

**H_1-a_.**  *The public operational resources (PORs) of health and wellness tourism have a significant positive influence on the RAC*.

**H_1-b_.**  *The infrastructures associated with health and wellness tourism exert a significant positive influence on the RAC*.

**H_1-c_.**  *The industry cooperation networks (ICNs) of health and wellness tourism exert a significant positive influence on the RAC*.

#### 2.2.2. Influences of RAC on II

Resources are a basic factor for industrial development. The II of health and wellness tourism depends on technological and information resources, labor resources, capital resources, and all kinds of material resources. The stronger the RAC is, the more abundant and the higher the quality of the resources obtained by the industry. This provides high-quality resource conditions for II and is conducive to shaping the competitive edge for the development of health and wellness tourism. Possession of valuable and scarce core resources is the foundation for the industry to shape its competitive edge. In an environment where the market competition becomes increasingly severe, various industries compete more fiercely for core resources. The industry forms its competitive edge and facilitates the II development by effectively identifying and acquiring external resources. Tourism enterprises’ constant acquisition and utilization of external resources, including knowledge [23], information, and capital [24], help to improve innovation ability and performance [19]. The following hypothesis is, therefore, proposed:

**H_2_.**  *The RAC of health and wellness tourism has a significant positive influence on the II*.

#### 2.2.3. Influences of IS on II

IS is an important basis and condition for the II development of health and wellness tourism. IS gathers the resources and basic elements needed for the II development of health and wellness tourism, to support the implementation of the II activities. Meanwhile, IS can radiate to and drive innovative development of relevant industries in the upstream and downstream of the industrial chain of health and wellness tourism. It fosters the II ability, forms an ecosystem, and promotes the II development of health and wellness tourism [25].

Natural resources, including landscape, air, water, local geological context, and climatic characteristics, are all the basis and condition for the innovation and development of health and wellness tourism. Differences in tourism resource endowment determine the location and attraction of core tourism products in a region [26]. The resources bring comparative advantages, and resource utilization produces the competitive edge, so resources and their utilization determine the innovation ability of health and wellness tourism [27]. Knowledge, as one of the most important innovation resources, plays an important part in facilitating II development. The more resources there are, such as information, management skills, and knowledge, the more conducive it is to improve the learning and innovation ability, thus obtaining high innovation performance [28]. Technical progress is the basis and power for the industrial structural reform of tourism. Technologies, particularly IT, have been extensively used in tourism and exert significant influences on tourism in innovating products, improving management efficiency, optimizing procedure, and reforming organizations. The development of IT and the Internet economy have induced innovation in the institution, operation and management, and market and changed the development mode of tourism [29]. Tourism is a labor-intensive industry, for which the human capital includes high-quality tourism professionals and entrepreneurs. The larger the scale is, and the higher the quality of the human capital is, the larger the number and the higher the quality of the innovation activities of tourism. Meanwhile, the efficiency and benefits of tourism will also be greatly improved, thus promoting the innovation and transformation of tourism [30].

Infrastructure is a key factor for the innovation and transformation of tourism, including traffic, transportation, post and telecommunications, and public service agencies. These infrastructures provide guarantee of traffic, knowledge and information, and public services for the innovation and development of tourism, thus promoting the innovation and transformation of tourism [31]. Research institutes and universities are sources of new technologies pertaining to tourism, provide knowledge and technical support for the II of tourism, and improve the technological level of tourism, thus facilitating the II development of tourism [32]. Shared network platforms, including O2O operation platforms, third-party payment platforms, and social platforms established based on modern IT such as Internet technology, big data, cloud computing, and blockchain, are links between innovation subjects. These II platforms combine innovation subjects inside and outside the industry, by using the coupling mechanism, and integrate, allocate, and share innovation resources. This, thus, realizes the efficient utilization of industrial resources and facilitates transformation and upgrading of the industrial structure [33].

Health and wellness tourism enterprises establish cooperative social networks with suppliers, distributors, dealers, and agents in the industrial chains as well as many organizations. These organizations include competitors, governments, industry associations, intermediary organs, and research institutes [34]. Health and wellness tourism enterprises obtain external innovation resources via these social networks, which form the advantages of resource agglomeration and economies of scale and, thus, promote innovation performance [35]. Establishing a cooperative relationship with suppliers helps these enterprises to obtain all sorts of resources such as research and development (R&D) technologies, management experience, market information, and new opportunities. Establishing benign cooperative relationships with the distributors, dealers, and agents of health and wellness tourism is advantageous for these enterprises to acquire resources needed, such as funds, market information, and technologies. It is also beneficial for resource sharing in the supply chain of health and wellness tourism, to improve the innovation ability of enterprises and facilitate II development [20]. Top-down and bottom-up industrial cooperation and the labor division and cooperative systems of supporting industries and relevant industries influence the II development [36]. Based on the above analysis, the following hypotheses are proposed:

**H_3-a_.**  *The PORs of health and wellness tourism exert a significant positive influence on the II*.

**H_3-b_.**  *The infrastructures for health and wellness tourism exert a significant positive influence on the II*.

**H_3-c_.**  *The ICNs of health and wellness tourism exert a significant positive influence on the II*.

#### 2.2.4. Mediating Effects of RAC

According to the above literature review, IS and its PORs, infrastructures, and ICNs directly influence the II on the one hand, regarding the influences of IS on the II of health and wellness tourism. To be specific, the IS of health and wellness tourism positively influences the II; in addition, the PORs [30], infrastructures [37], and ICNs [35] exert positive influences on the II. On the other hand, the IS of health and wellness tourism and its PORs, infrastructures, and ICNs indirectly affect the II via the RAC. To be specific, the IS of the industry positively influences the RAC; besides, the PORs [16], infrastructures [38], and ICNs positively influence the RAC. Meanwhile, the RAC positively affects the II. Therefore, the following hypotheses are proposed:

**H_4-a_.**  *The RAC serves as a partial mediating variable between the PORs and II of health and wellness tourism*.

**H_4-b_.**  *The RAC serves as a partial mediating variable between the infrastructures and II of health and wellness tourism*.

**H_4-c_.**  *The RAC serves as a partial mediating variable between the ICNs and II of health and wellness tourism*.

#### 2.2.5. Moderating Effects of Policy Support

In the Chinese context, government and policy support (PS) plays an important part in the II development and, therefore, is a key factor that affects the II development of health and wellness tourism [39]. The research indicates that the IS of health and wellness tourism and its PORs, infrastructures, and ICNs have significant influences on the II. However, the PORs, infrastructures, and ICNs differ in terms of their degrees of influence. This indicates that a certain moderating variable plays a role between the constitutional dimensions of IS (PORs, infrastructures, and ICNs) and the II. Tourism has many associations and involves a wide range of industries, in which the government plays a role as planner, coordinator, and driver in the development of tourism [40]. The government controls many policy tools for promoting innovation and motivates the II activities in aspects including financial support, preferential tax, fiscal subsidies, and innovation awards, by formulating preferential policies. This is conducive to providing those resources needed for innovation [41]. A series of industrial support policies launched by the government contributes to the creation of a favorable industrial environment. This can also attract the investment of social capital, to obtain external funds [39]. Additionally, this attracts lots of tourism enterprises and outstanding tourism professionals to aggregate [26], to motivate industrial vitality and creativity, and to influence the innovation ability of the industry. The macro-control policies formulated by the government can also facilitate infrastructure construction, urban planning, and ecological environmental protection and promote the innovation and transformation of tourism.

Based on the above analysis, the following hypotheses are proposed:

**H_5-a_.**  *The PS exerts a moderating effect between the PORs and II of health and wellness tourism. That is, the PORs of health and wellness tourism, under greater government support, contribute to greater improvement in the II, compared with those under lower government support*.

**H_5-b_.**  *The PS exerts a moderating effect between the infrastructures and II of health and wellness tourism. This means that the infrastructures of health and wellness tourism, under greater government support, lead to greater improvement in the II, compared with those under lower government support*.

**H_5-c_.**  *The PS has a moderating effect between the ICNs and II of health and wellness tourism, i.e., the ICNs of health and wellness tourism, under greater government support, lead to more substantial improvement in the II, compared with those under lower government support*.

All hypotheses can be seen in Table 1.

### 2.3. Conceptual Model

**Figure 1:**
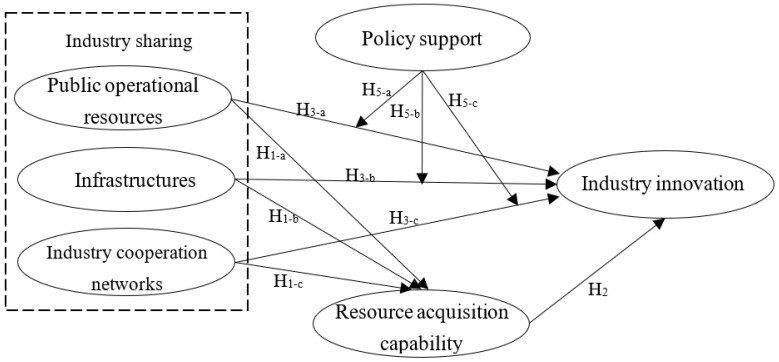
Conceptual model.

The development mechanism of the II of the industry, in the context of sharing economy, is established (Figure 1). This is based on the above theoretical analysis and hypotheses for relationships of IS and its constitutional dimensions (PORs, infrastructures, and ICNs) with the II variables of health and wellness tourism; therein, the RAC functions as a mediating variable, and the PS serves as a moderating variable.

## 3. Research Design

### 3.1. Variable Measurements

#### 3.1.1. IS

By using modern ITs, including the Internet, IS mainly features sharing of the right of use. This optimizes the allocation and integration of a set of core resources inside and outside health and wellness tourism, forming a set of common resources supporting the II development of the industry. By referring to scales used by Laesser (2011) [42] and Yuan (2013) [43], 14 items are used for the IS of health and wellness tourism, which belong to three constitutional dimensions: PORs (6 items, including natural ecological environment, medical institutions, sports facilities, professional and technical talents, technical resources, and tourism brand), infrastructures (4 items, including research institutions, network platform, tourism infrastructure, and commercial service facilities), and ICNs (4 items, including mature industry cooperation alliance, cooperation efficiency with upstream suppliers, cooperation efficiency with downstream dealers, and cooperation efficiency with relevant auxiliary industries).

#### 3.1.2. PS

PS refers to the direct and indirect promotion effects on the industry under a series of laws and regulation as well as the policies and guidelines formulated by the national and regional governments. Three items are used for PS, by referring to the scales designed by Veciam and Urbano (2008) [44] and Xu (2018) [45], including “the government formulates the development plan of the industry”, “the government strongly supports the development of health and wellness tourism industry”, and “the government has preferential policies for tourism enterprises”.

#### 3.1.3. RAC

RAC means the ability of organizations or individuals in acquiring useful resources. The research refers to the scales formulated by Wiklund (2003) [46] and Li and Xu (2019) [38]. Three items are employed for RAC, including “enterprises in the industry can conveniently obtain needed operational resources from the industry”, “enterprises can conveniently obtain market information from the industry”, and “enterprises can conveniently obtain funds, facilities and equipment from the industry”.

#### 3.1.4. II

II is a process to form new production capacity and improve the competitiveness of health and wellness tourism, by introducing new combinations of new production factors and conditions into the production systems of the industry using a series of methods and approaches. These approaches include technological R&D, market development, organizational restructuring, and product innovation. By referring to the scales formulated by Yeoh (2009) [47] and Gao (2017) [19], four items are included for II, including “new products and services are frequently launched in the industry”, “the sales revenue of new products in the industry is rising”, “the market share of the industry is gradually expanding”, and “the production efficiency of the industry is gradually improved”.

All of the 24 variables above were measured on a Likert scale from 1 to 5 (1 = totally disagree, and 5 = totally agree).

### 3.2. Data Collection

The respondents were the operators and managers of health and wellness tourism enterprises. The survey was conducted from March to May 2020. The researchers wrote letters to the tourism commissions of some provinces to seek their cooperation and, finally, had the support of 21 provinces (or municipalities) in China. These include Shanghai; Chongqing; Beijing; Tianjin; Heilongjiang; Hebei; Henan; Shandong; Shanxi; Anhui; Hubei; Jiangsu; Sichuan; Guizhou; Yunnan; Guangxi; Zhejiang; Jiangxi; Guangdong; Fujian; and Hainan. With their help; researchers contacted the managers of health and wellness tourism enterprises to conduct an investigation, by mailing paper questionnaires and online questionnaires. Before any formal investigation, the researchers introduced the investigation procedures and methods to the persons in charge of the enterprises. A total of 710 questionnaires were released and 621 were returned, so the recovery rate of questionnaires was 87.5%. Through screening, 79 questionnaires were eliminated due to incompleteness and low authenticity, so 542 valid questionnaires were obtained (Table 2). The valid questionnaire rate was 87.3%.

### 3.3. Research Methods

Statistical software SPSS19.0 was employed to conduct reliability analysis and factor analysis on the scales of each variable, to test the reliability and construct validity of each scale. AMOS17.0 software was adopted for the path analysis of the relationship model between IS and II, to test the hypotheses for the path relationships of each variable. In addition, stratified regression analysis was used to test the moderating effect of PS.

## 4. Results and Analysis

### 4.1. Reliability and Validity Tests of Scales

In the reliability test, the statistical software SPSS19.0 was used for the reliability analysis of scales of each variable. The results show that the Cronbach *α* coefficients of scale of the constitutional dimensions (PORs, infrastructures, and ICNs), RAC, PS, II, and IS are 0.838, 0.849, 0.905, 0.877, 0.910, 0.896, and 0.903, respectively, which are all greater than 0.8. The results indicate that the internal consistency reliability of scales of each variable meets the requirements.

In terms of validity tests, the statistical software SPSS19.0 was adopted for the factor analysis of the scales of each variable. The results show that the relevant indices of each scale are all acceptable according to standards (Table 3). The Kaiser–Meyer–Olkin (KMO) values of each scale are greater than 0.7, and the *p* value in Bartlett sphericity tests is 0.000, indicating that each scale is suitable for factor analysis (Kaiser, 1974) [48]. After orthogonal rotation (varimax), the number of common factors with the extracted eigenvalues greater than 1 for items of each variable are consistent with the psychological traits of the theoretical structure. The factor loading is always greater than 0.50, and the total variance explained is always above the requisite standard of 70%. These results indicate that the scales of each variable have favorable construct validity. Regarding content validity, the items in each scale are designed after a wide literature review and are evaluated and revised by experts, on the basis of referring to mature measuring tools from around the world. This ensures that the items in the scales appropriately represent the test content; therefore, each scale has high content validity.

### 4.2. Correlation Analysis

Correlation analysis was conducted on the variables IS, PS, and RAC with the II, using SPSS19.0 software. The results (Table 4) indicate that IS, PS, and RAC are significantly correlated with II at the significance level of 0.01 (two-tailed). Therefore, the above variables have correlations. However, the results fail to reveal the extent of the influences and causality among variables, so correlations of various variables need to be tested further using the structural equation model.

### 4.3. Test Results of the Mediating Effect of RAC

AMOS17.0 software was adopted to determine the paths of the structural equation for the mediating effect of the RAC for all 542 samples in the formal survey. Results indicate that, in terms of the overall fitting index of the model, the various indices are within the ranges of corresponding critical values. In addition, the model is a good fit to the sample data, showing the effectiveness and reasonability of the overall structure. The path analysis results of the structural equation model are summarized in Table 5.

According to the test results of the model for the relationships between the IS and II of health and wellness tourism using AMOS17.0, the direct, indirect, and total effects of the variables, including the PORs, infrastructures, ICNs, and RAC with the II of the industry, were calculated (Table 6).

#### 4.3.1. Influences of PORs on RAC and II

The direct effect of the PORs on the RAC of health and wellness tourism is 0.425 (*p* < 0.001), suggesting that the former has a significant positive influence on the latter, and, therefore, hypothesis H_1-a_ is true. The direct, indirect, and total effects of the PORs on the II of health and wellness tourism are 0.238 (*p* < 0.05), 0.099 (*p* < 0.001), and 0.337, respectively, indicating that the PORs have significant positive influences on the II, so hypothesis H_3-a_ is tenable. Since the PORs not only directly positively influence the II but also indirectly positively influence the II via the RAC, the RAC serves as a partial mediating variable between the PORs and II of health and wellness tourism. Therefore, hypothesis H_4-a_ holds.

#### 4.3.2. Influences of Infrastructures on RAC and II

The infrastructures associated with health and wellness tourism exert a direct influence of 0.165 (*p* < 0.05) on the RAC, indicating that the infrastructures have a significant positive influence on the RAC, and hypothesis H_1-b_ is true. The direct, indirect, and total effects of infrastructures on the II of health and wellness tourism are 0.338 (*p* < 0.001), 0.038 (*p* < 0.001), and 0.376, respectively, suggesting that the infrastructures significantly positively affect II, so hypothesis H_3-b_ is tenable; since the infrastructures not only directly positively influence the II but also indirectly positively affect the II through the RAC, the RAC functions as a partial mediating variable between the infrastructures and II of health and wellness tourism, and hypothesis H_4-b_ is true.

#### 4.3.3. Influences of ICNs on RAC and II

The direct effect of the ICNs on the RAC of health and wellness tourism is 0.441 (*p* < 0.001), which implies that the ICNs have a significant positive influence on the RAC, and hypothesis H_1-c_ holds. The direct, indirect, and total effects of the ICNs on the II of health and wellness tourism are 0.173 (*p* < 0.05), 0.102 (*p* < 0.001), and 0.275, respectively, indicating that the ICNs significantly positively affect the II, and hypothesis H_3-c_ is true. In addition, the RAC serves as a partial mediating variable between the ICNs and II of health and wellness tourism, because the ICNs not only directly positively affect the II but also indirectly positively affect the II via the RAC. Therefore, hypothesis H_4-c_ is tenable.

#### 4.3.4. Influences of RAC on II

The direct effect of the RAC on the II of health and wellness tourism is 0.232 (*p* < 0.001), which implies that the RAC exerts a significant positive influence on the II, and hypothesis H_2_ is true.

According to the above analysis, hypotheses H_1_ to H_4_ (Table 1) for the relationships of the various variables in the model for the influencing mechanism of IS on the II of health and wellness tourism established in the research have all been tested and found to be true. In terms of the II, the infrastructures of IS have the greatest influence on the II (0.376), followed successively by the PORs (0.337), ICNs (0.275), and RAC (0.232), from the total effects of the various variables. For the RAC, the ICNs of the IS of health and wellness tourism exert the greatest influences on the RAC (0.441), followed by the PORs (0.425) and infrastructures (0.165).

### 4.4. Test Results of the Moderating Effect of PS

SPSS19.0 software was used for hierarchical regression analysis of the PORs, infrastructures, and ICNs of IS as well as the PS and II of health and wellness tourism. The criterion for judging the moderating variable to have a significant moderating effect is that the coefficient of the interaction terms in the regression model is significantly non-zero. To avoid multi-collinearity among variables, centralized transformation (subtracting the means of variables) was performed separately for the independent variables (PORs, infrastructures, and ICNs) and the moderating variable (PS).

#### 4.4.1. Tests of the Moderating Effect of PS between PORs and II

SPSS19.0 software was adopted for hierarchical regression analysis of the II for the PORs and PS, and the results are shown in Table 7. The variance contributions of the PS and PORs to the II are 44.4% and 31.0%, respectively, and the interaction term between the PS and PORs has a variance contribution of 0.5% to the II, all of which have passed the *F*-test (significant at the *p* < 0.001 level). The coefficient *β* of the interaction term of the PORs × PS is −0.079 (t = −2.297, *p* < 0.05), which is significantly non-zero, indicating that the PS exerts a moderating effect between the PORs and II, so hypothesis H_5-a_ is found to be true. The tolerability and variance inflation factor (VIF) are used for the multi-collinearity tests of the regression equation. Results show that the tolerability of each independent variable is always greater than 0.1, and its VIF is always lower than 10 (Wu, 2010), which suggests that the independent variables in the regression equation do not exhibit any significant multi-collinearity.

#### 4.4.2. Test of the Moderating Effect of PS between Infrastructures and II

Hierarchical regression analysis was conducted on the II for infrastructure and PS using SPSS19.0 software, and the results are listed in Table 8. The variance contributions of the PS and infrastructures to the II are 44.4% and 31.2%, respectively, and the variance contribution of the interaction term between the PS and infrastructures to the II is 0.5%, all passing the *F*-test (significant at the *p* < 0.001 level). The coefficient *β* of the interaction term of the infrastructures × PS is −0.081 (t = −3.186, *p* < 0.05), which is significantly non-zero, indicating that the PS has a moderating effect between the infrastructures and II, so hypothesis H_5-b_ is tenable. Then, the tolerability and VIF are adopted for multi-collinearity testing of the regression equation. The results reveal that the various variables all have tolerability greater than 0.1 and VIF lower than 10, which implies that the independent variables in the regression equation do not exhibit any significant multi-collinearity.

#### 4.4.3. Test of the Moderating Effect of PS between ICNs and II

Hierarchical regression analysis was performed on the II for the ICNs and PS using SPSS19.0 software, and the results are displayed in Table 9. The PS and ICNs have variance contributions of 44.4% and 31.4%, respectively, to the II, and the variance contribution of the interaction term between the two to the II is 1.0%, all passing the *F*-test (significant at the *p* < 0.001 level). The interaction term of the ICNs × PS has a coefficient *β* of −0.117 (t = −4.781, *p* < 0.001), which is significantly non-zero. This indicates that the PS has a moderating effect between the ICNs and II, and, therefore, hypothesis H_5-c_ is found to be true. Thereafter, the tolerability and VIF are used to conduct a multi-collinearity test. The results show that the various independent variables all have tolerability greater than 0.1 and VIF lower than 10, which means that there is no significant multi-collinearity among the independent variables in the regression equation.

In summary, the PS exerts a positive moderating effect between the PORs and II of health and wellness tourism (H_5-a_ is true). The PS has a positive moderating effect between the infrastructures and II of health and wellness tourism (H_5-b_ is tenable). The PS has a positive moderating effect between the ICNs and II of health and wellness tourism (H_5-c_ is tenable). Therefore, the PS serves as a moderating variable between the IS and II of health and wellness tourism.

## 5. Conclusions and Discussion

### 5.1. Conclusions

(1) The three constitutional dimensions of the IS of health and wellness tourism, namely, PORs, infrastructures, and ICNs, exert significant positive influences on II, i.e., IS has a significant positive influence on II. The study finds that in the sharing economy environment, the three components of industry sharing are the key factors that influence the development of the industry innovation of health and wellness tourism industry. Among them, infrastructures have the most significant influences on II (total effect is 0.376), followed successively by PORs (total effect is 0.337) and ICNs (total effect is 0.275). 

(2) RAC exerts a mediating effect between the IS and II of health and wellness tourism. PORs, infrastructures, and ICNs of the IS of health and wellness tourism not only directly positively influence II but also indirectly positively affect II via RAC. Therefore, RAC functions as a partial mediating variable between IS and II. 

(3) PS plays a moderating role between the IS and II of health and wellness tourism. PS always exerts a positive moderating effect between the various dimensions of IS (PORs, infrastructures, and ICNs) and II. Therefore, PS serves as a moderating variable between the IS and II of health and wellness tourism.

Based on the above analysis, in the sharing economy environment, the components of industry sharing (PORs, infrastructures, and ICNs), on the one hand, directly and positively affect II, and, on the other hand, indirectly and positively affect II through RAC. At the same time, PS plays a moderating role between the PORs, infrastructures, ICNs, and II of the health and wellness tourism industry. Therefore, hypotheses H_1_ to H_5_ (Table 1) for the relationships of the various variables proposed in the model for the influencing mechanism of IS on the II of health and wellness tourism have been tested and found to be tenable.

### 5.2. Theoretical and Practical Inspiration

#### 5.2.1. Theoretical Inspiration

(1) Studying the influencing mechanisms of the II of health and wellness tourism from the perspective of IS provides a new theoretical perspective for research on the II of the industry. It is also favorable for filling the gap of study from the interdisciplinary theoretical perspective and deepening the existing theoretical research. Existing research findings are limited to the theory of a traditional industrial economy. However, there is still a lack of research findings about the II development of health and wellness tourism, from the perspective of combining theories of the sharing economy and the traditional industrial economy. The research extends the influencing mechanism of the II of health and wellness tourism from the traditional industrial economy to the IS perspective. In addition, the influencing mechanisms of the constitutional dimensions of the IS of health and wellness tourism, including PORs, infrastructures, and ICNs, on II are elucidated. This research not only enriches the research on the constitutional dimensions and measurement indices of the IS of health and wellness tourism and provides a tool for future research but also expands the interdisciplinary research on the sharing economy and II. 

(2) RAC has a partial mediating effect between the IS and II of health and wellness tourism. Although existing research findings in the academic community have confirmed that RAC is positively correlated with II, there is still a dispute about the mediating effect of RAC on II. The current research verifies that RAC exerts a partial mediating effect in the model for the influencing mechanism of IS on the II of health and wellness tourism. The conclusion differs from the point of view of other scholars: that RAC is only an antecedent variable. In the context of the sharing economy, the growth of health and wellness tourism enterprises breaks through the constraint of traditional endogenous resources, and, instead, the growth is exogenous; these enterprises are resource assemblies in the IS framework, where the internal and external resources of enterprises jointly determine their growth scale and quality. IS gathers the resources and basic factors needed for the II development of health and wellness tourism, thus supporting the implementation of II activities. In the context of the sharing economy, the IS of health and wellness tourism influences RAC and, further, II, so RAC has a partial mediating effect between the IS and II of the industry. This differs, to some extent, from traditional enterprise growth theory and shows the exogeneity of the growth of health and wellness tourism enterprises in the context of the sharing economy. 

(3) The introduction of the PS variable helps to clarify the boundary of the effects of policies in the Chinese context on the innovative development of health and wellness tourism enterprises. In the existing literature, policies (governments) are mainly regarded as an antecedent variable of the II, and the effect of PS on IS and II is seldom studied. Health and wellness tourism is characterized by large investment scale, its long investment return cycle, high capital risk, and significant difficulty in attracting investment. On the Chinese market, health and wellness tourism is still in its initial stage, so PS has become an important factor influencing the sustainable development of the industry. In the research, PS is introduced as a moderating variable, and it is empirically verified that PS serves as a moderating variable between the constitutional dimensions of IS (PORs, infrastructures, and ICNs) and II. This highlights the localized features of the research model and further enriches the understanding of the academic community about the nature of the effects of PS on II.

#### 5.2.2. Practical Inspiration

(1) Tourism destinations should establish the concept of industry innovation from the perspective of industry sharing. Some places only focus on partial factors in the process of developing health and wellness tourism industry, which will easily produce a “short board effect” and eventually affect the sustainable development of the health and wellness tourism industry. Tourism destinations should comprehensively apply management means to improve the industry sharing level of the health and wellness tourism industry as a whole and improve the level of PORs, infrastructures, and ICNs at the same time, without ignoring any of them. 

(2) Improving the level of PORs, infrastructures, and ICNs can help to enhance the industry sharing of the health and wellness tourism industry. In terms of PORs, tourism destinations should make comprehensive use of policies and market means to guide large Internet enterprises and tourism enterprises to develop network platforms, which can realize resource sharing of capitals, talents, materials, technologies, information, etc. In terms of infrastructures, it is necessary to improve barrier-free facilities in scenic spots, hotels, tourist vehicles, public restrooms, and other places. In terms of ICNs, government regulation and market means should be used to build tourism enterprise groups, industry clusters, industrial parks, etc. The health and wellness tourism industry should strengthen the integration with sports, medical care, culture, information, education, and other industries, so as to form a networked industry ecosystem.

(3) PS is conducive to promoting innovation in the health and wellness tourism industry. Tourism destinations should establish a perfect policy system to strengthen financial support for tourism enterprises and give more preferential policies to them in investment, approval, taxation, loans, etc. The government should deepen the marketization reform of rehabilitation and healing institutes. Preferential policies should be used to attract private capital and foreign capital into the health and wellness tourism industry, so as to reduce the proportion of the state-owned economy.

## Figures and Tables

**Table 1:** Summary of hypotheses.

Serial No.	Hypothesis
H_1-a_	*PORs of health and wellness tourism exert a significant positive influence on RAC.*
H_1-b_	*Infrastructures of health and wellness tourism exert a significant positive influence on RAC.*
H_1-c_	*ICNs of health and wellness tourism exert a significant positive influence on RAC.*
H_2_	*RAC of health and wellness tourism has a significant positive influence on II.*
H_3-a_	*PORs of health and wellness tourism have a significant positive influence on II.*
H_3-b_	*Infrastructures of health and wellness tourism have a significant positive influence on II.*
H_3-c_	*ICNs of health and wellness tourism have a significant positive influence on II.*
H_4-a_	*RAC serves as a partial mediating variable between PORs and II of health and wellness tourism.*
H_4-b_	*RAC serves as a partial mediating variable between infrastructures and II of health and wellness tourism.*
H_4-c_	*RAC serves as a partial mediating variable between ICNs and II of health and wellness tourism.*
H_5-a_	*PS exerts a moderating effect between PORs and II of health and wellness tourism.*
H_5-b_	*PS exerts a moderating effect between infrastructures and II of health and wellness tourism.*
H_5-c_	*PS has a moderating effect between ICNs and II of health and wellness tourism.*

**Table 2:** Demographic profile of respondents.

Indices	Items	Numbers	Percentage
Gender	Female	244	45.0
	Male	298	55.0
Age	18–29	121	22.3
	30–39	207	38.2
	40–49	156	28.8
	50–59	58	10.7
Education background	High school	86	15.9
	Junior college education	193	35.6
	Undergraduate	238	43.9
	Postgraduate	25	4.6
Title	Supervisor	251	46.3
	Department manager	157	29.0
	Deputy general manager	75	13.8
	General manager	59	10.9

**Table 3:** Output of factor analysis.

Variables	KMO	Sig.	Factors	Cronbach’s	Total Variance Explained
IS	0.815	0.000	3	0.903	77.511%
RAC	0.739	0.000	1	0.878	80.382%
PS	0.750	0.000	1	0.910	84.705%
II	0.825	0.000	1	0.898	76.549%

**Table 4:** Descriptive statistical analysis and correlation matrix of each variable.

Variables	Mean	Standard Deviation	IS	PS	RAC	II
IS	3.70368	0.794871	1	0.214 **	0.668 **	0.595 **
PS	3.32016	0.904558	0.214 **	1	0.272 **	0.666 **
RAC	3.78454	0.810669	0.668 **	0.272 **	1	0.648 **
II	3.78571	0.756257	0.595 **	0.666 **	0.648 **	1

** Means *p* < 0.01.

**Table 5:** Influence of IS on II of health and wellness tourism industry.

			Estimate	S.E.	C.R.	*p*
RAC	←	PORs	0.425	0.101	4.216	***
RAC	←	Infrastructures	0.165	0.070	2.351	*
RAC	←	ICNs	0.441	0.059	7.476	***
II	←	RAC	0.232	0.057	4.086	***
II	←	PORs	0.238	0.092	2.571	*
II	←	Infrastructures	0.338	0.065	5.204	***
II	←	ICNs	0.173	0.057	3.035	*

*** Means *p* < 0.001, * Means *p* < 0.05.

**Table 6:** Effect size of variables.

Outcome Variables	Antecedent Variable	Direct Effect	Indirect Effect	Total Effect
RAC	PORs	0.425	-	0.425
	Infrastructures	0.165	-	0.165
	ICNs	0.441	-	0.441
II	PORs	0.238	0.099	0.337
	Infrastructures	0.338	0.038	0.376
	ICNs	0.173	0.102	0.275
	RAC	0.232	-	0.232

- Means there is no indirect effect.

**Table 7:** Hierarchical regression analysis of relationship between PORs, PS, and II.

Variables	Dependent Variable: II		
	Model 1	Model 2	Model 3
Constant	3.784 ***	3.784 ***	3.786 ***
PS	0.558 ***	0.543 ***	0.551 ***
PORs	-	0.674 ***	0.667 ***
PORs × PS	-	-	−0.079 *
R^2^	0.444	0.754	0.757
F	431.747 ***	827.627 ***	557.885 ***
△R^2^	0.444	0.310	0.005
△F	431.747 ***	680.348 **	5.275 **

*** Means *p* < 0.001, ** Means *p* < 0.01, * Means *p* < 0.05.

**Table 8:** Hierarchical regression analysis of relationship between infrastructures, PS, and II.

Variables	Dependent Variable: II		
	Model 1	Model 2	Model 3
Constant	3.784 ***	3.785 ***	3.792 ***
PS	0.558 ***	0.497 ***	0.515 ***
Infrastructures	-	0.580 ***	0.574 ***
Infrastructures * PS	-	-	−0.081 *
R^2^	0.444	0.757	0.761
F	431.747 ***	837.377 ***	571.111 ***
△R^2^	0.444	0.312	0.005
△F	431.747 ***	691.184 ***	10.150 **

*** Means *p* < 0.001, ** Means *p* < 0.01, * Means *p* < 0.05.

**Table 9:** Hierarchical regression analysis of relationship between ICNs, PS, and II.

Variables	Dependent Variable: II		
	Model 2	Model 2	Model 2
Constant	3.784 ***	3.785 ***	3.797 ***
PS	0.558 ***	0.485 ***	0.506 ***
ICNs	-	0.557 ***	0.555 ***
ICNs * PS	-	-	−0.117 ***
R^2^	0.444	0.758	0.768
F	431.747 ***	846.250 ***	594.668 ***
△R^2^	0.444	0.314	0.010
△F	431.747 ***	701.045 ***	22.860 **

*** Means *p* < 0.001, ** Means *p* < 0.01.

## Data Availability

The data presented in this study are available on request from the corresponding author.
